# Sensitive and Precise Quantification of Insulin-Like mRNA Expression in *Caenorhabditis elegans*

**DOI:** 10.1371/journal.pone.0018086

**Published:** 2011-03-22

**Authors:** L. Ryan Baugh, Nicole Kurhanewicz, Paul W. Sternberg

**Affiliations:** 1 Department of Biology and IGSP Center for Systems Biology, Duke University, Durham, North Carolina, United States of America; 2 Howard Hughes Medical Institute and Division of Biology, California Institute of Technology, Pasadena, California, United States of America; Buck Institute for Age Research, United States of America

## Abstract

Insulin-like signaling regulates developmental arrest, stress resistance and lifespan in the nematode *Caenorhabditis elegans*. However, the genome encodes 40 insulin-like peptides, and the regulation and function of individual peptides is largely uncharacterized. We used the nCounter platform to measure mRNA expression of all 40 insulin-like peptides as well as the insulin-like receptor *daf-2*, its transcriptional effector *daf-16*, and the *daf-16* target gene *sod-3*. We validated the platform using 53 RNA samples previously characterized by high density oligonucleotide microarray analysis. For this set of genes and the standard nCounter protocol, sensitivity and precision were comparable between the two platforms. We optimized conditions of the nCounter assay by varying the mass of total RNA used for hybridization, thereby increasing sensitivity up to 50-fold and reducing the median coefficient of variation as much as 4-fold. We used deletion mutants to demonstrate specificity of the assay, and we used optimized conditions to assay insulin-like gene expression throughout the *C. elegans* life cycle. We detected expression for nearly all insulin-like genes and find that they are expressed in a variety of distinct patterns suggesting complexity of regulation and specificity of function. We identified insulin-like genes that are specifically expressed during developmental arrest, larval development, adulthood and embryogenesis. These results demonstrate that the nCounter platform provides a powerful approach to analyzing insulin-like gene expression dynamics, and they suggest hypotheses about the function of individual insulin-like genes.

## Introduction

Insulin-like signaling contributes to homeostasis in multi-cellular animals by mediating physiological responses to environmental conditions through systemic signaling. In mammals, insulin signaling regulates carbohydrate metabolism, and insulin-like growth factor signaling controls growth. In invertebrates, insulin-like signaling regulates growth and metabolism as well as other aspects of developmental physiology [1]. In the nematode *C. elegans*, insulin-like signaling regulates formation of a stress resistant, non-feeding developmental alternative to the third larval stage known as the dauer larva [2]. Dauers form in conditions that are not favorable for growth and reproduction, and they serve as a dispersal mechanism. Dauer formation is triggered primarily in response to high population density but also limiting food and high temperature [3]. Insulin-like signaling also regulates an acute form of developmental arrest that occurs in response to complete starvation (L1 arrest) [4], [5]. Insulin-like signaling regulates adult lifespan in *C. elegans* [6], [7], [8], [9], as well as the fly *Drosophila melanogaster* and mammals [10], [11], [12].

Insects and nematodes each have several insulin-like peptides, and relatively little is known about the function of specific peptides [1]. The *C. elegans* genome encodes 40 putative insulin-like peptides [13]. The extent to which individual peptides have overlapping vs. specific function is not understood, and the complexity of the signaling network they comprise is unclear. Insulin-like gene expression is transcriptionally controlled in *C. elegans* [14], and expression analysis offers a way to infer the dynamics of the insulin-like signaling network in response to varying environmental conditions. A subset of insulin-like genes have been analyzed by transcriptional reporter genes, but conditional regulation was not investigated, expression was not quantified, and dynamics were not analyzed [13]. Measurement of endogenous mRNA is ideal but challenging since insulin-like genes are expressed at relatively low levels in whole worms. Furthermore, microarrays produced to date measure only about half of the 40 insulin-like genes, and comprehensive QPCR analysis has not been reported.

nCounter is a commercially available platform for mRNA expression analysis [15]. The nCounter “code set” contains a pair of ∼50 nt biotinylated DNA probes that are barcoded with different combinations of fluorescent tags. Total RNA is hybridized with the code set in solution phase, and DNA:mRNA hybrids are captured on the surface of a flow-cell, stretched by an electric field, and imaged. Fluorescent tags are optically resolved so that barcodes can be read and counted. Compared to other technologies for mRNA expression analysis, sensitivity should benefit from solution phase hybridization, whereas counting mRNA molecules should aid precision. In addition, the system has the benefit of measuring total RNA directly, avoiding biases potentially introduced by the use of enzymes or amplification. Although the approach is not genome-wide, accommodating tens to hundreds of genes per code set, the ease with which samples can be processed makes it excellent for experiments measuring expression over many time points, conditions or genotypes [16].

The objectives of this study were to determine the feasibility of using the nCounter platform for insulin-like mRNA expression analysis in *C. elegans* and to provide an overview of insulin-like gene expression. This goal includes validating the platform against the Affymetrix microarray platform, benchmarking and optimizing sensitivity and precision, and using the platform to measure insulin-like gene expression during the *C. elegans* life cycle. The results show that the platform produces reliable expression measurements with unparalleled sensitivity and precision. We also found that each of the insulin-like genes measured is expressed, and their expression patterns are largely distinct, suggesting that their regulation is complex leading to specific albeit overlapping functions.

## Results and Discussion

### Platform comparison between nCounter and microarray analysis

**Figure 1:**
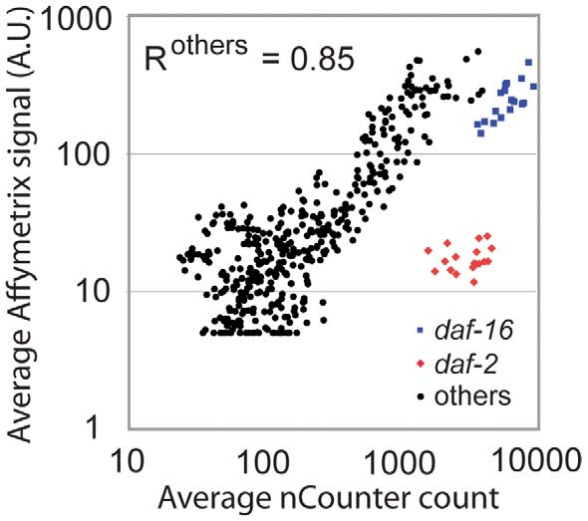
Validation of the nCounter platform with Affymetrix microarray analysis. 0.1 µg total RNA from 53 independent RNA preparations was used for nCounter analysis. The 53 samples comprise 18 groups of biological replicates (all but one with 3 replicates), and the average of each group is plotted for 25 genes common to both platforms. Each axis is on a log scale.

We used total RNA samples previously characterized by Affymetrix microarray analysis to test the nCounter platform [17]. We purchased an nCounter code set from NanoString, Inc. that included probes for all 40 *C. elegans* insulin-like genes as well as the insulin-like receptor *daf-2* [7], its transcriptional effector *daf-16* [8], [9], and the *daf-16* target gene *sod-3* [18]. The microarray contained probe sets for 22 of the insulin-like genes, as well as *daf-2*, *daf-16*, and *sod-3*. For microarray analysis 0.1 µg total RNA was used for biotin-labeled cRNA synthesis; for nCounter analysis 0.1 µg total RNA was used directly for hybridization. 53 RNA samples were analyzed on each platform. The experimental design included 18 groups of biological replicates, and the average of each group was compared for the 25 genes common to both platforms (Figure 1).

nCounter analysis of mRNA expression agreed well with microarray analysis (Figure 1). There was some quantitative disagreement between the platforms, but it was relatively minor and evenly distributed. The exceptions were *daf-2* and *daf-16*. Both genes are large with multiple splice forms, and the two platforms did not target the same transcript sequences. *daf-2* was not detected above background by microarray, but it was robustly detected by nCounter. *daf-16* was detected with greater sensitivity by nCounter than microarray, but the expression pattern was qualitatively similar between the two (data not shown). Excluding *daf-2* and *daf-16*, the correlation coefficient for the platform comparison was 0.85. We believe this is very good agreement considering the technical differences between the two procedures and the fact that the insulin-like genes are expressed at relatively low levels in whole worms. For this set of genes and this amount of RNA (0.1 µg) the sensitivity of the two platforms is comparable based on the number of genes detected above background. The nCounter results are modestly more reproducible with a median coefficient of variation of 17% compared to 25% for the microarray results, considering the same set of genes in each case. In summary, the nCounter platform performed well, matching or exceeding Affymetrix microarray analysis in terms of sensitivity and precision using the same mass of total RNA as starting material.

### Optimization of total RNA mass used in nCounter hybridization

**Figure 2:**
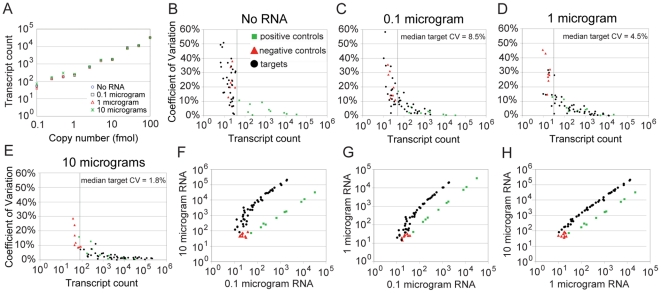
Code set specific optimization of total RNA mass used in nCounter hybridization increases sensitivity and precision. (A) Positive control standard curve is plotted for each RNA mass. Average transcript count (3 technical replicates) is plotted against the coefficient of variation for (B) no RNA, (C) 0.1 µg, (D) 1 µg, and (E) 10 µg. (F–H) Average transcript count is plotted in 3 pair-wise comparisons of the 3 RNA masses used. Legend in B applies to B–H. The y-axis of B–E is coefficient of variation. The vertical grey line in B–E represents background. Data are normalized by positive control counts. Transcript counts are plotted on a log scale. “CV” refers to coefficient of variation. One data point is omitted from B (a target with count of 57 and CV of 120%).

We performed an experiment to analyze the effects of total RNA mass used for hybridization to the nCounter code set on sensitivity and precision. The standard nCounter protocol specifies 0.1 µg total RNA for hybridization. However, our code set contained probes for only 43 genes, which is less than one-tenth of what the system can accommodate. In addition, the insulin-like genes are expressed at relatively low abundance. As a result, we typically observed less than 100,000 total counts per hybridization using 0.1 µg total RNA, though the system should allow for millions of total counts. This motivated us to try using more RNA per hybridization, and to investigate the effects of using no RNA as a control. We performed a set of technical replicates, all from a single total RNA preparation. We used no RNA, 0.1 µg, 1 µg and 10 µg total RNA in otherwise identical hybridizations. For each mass of RNA we performed 3 replicates. The code set includes a set of 10 positive controls. The positive controls include sequences from *A. thaliana* and *D. melanogaster* (File S1), and synthetic transcripts complementary to each were included in the code set at known concentrations. The standard curve resulting from positive control counts was not affected by total RNA mass (Figure 2A). We therefore used the sum of positive control counts to normalize the data across all 12 samples.

Sensitivity was improved by increasing the RNA mass used in hybridization. The average number of counts obtained for target transcripts (43 genes) was approximately 10-fold higher with 1 µg compared to 0.1 µg RNA and approximately 100-fold higher with 10 µg (Table 1). However, background increased with 10 µg RNA, as indicated by an approximate doubling in the average number of counts obtained for negative controls (*A. thaliana* and *D. melanogaster* probes; Table 1 and File S1). Consistent with the number of counts increasing, we also detected more target genes with more hybridized RNA. Background was modeled for each mass of RNA as the average of negative control transcript counts plus three standard deviations. Based on this background model, none of the negative controls were detected in any of the hybridizations, and only one of the targets was detected when no RNA was used for hybridization (Table 2). Over 1.5 times as many targets were detected with 1 µg RNA compared to 0.1 µg, and all but one of the 43 targets were detected with 10 µg. However, consistent with background increasing with more hybridized RNA, the lowest abundance positive control was not detected above background with 1 µg or 10 µg RNA (Table 2). This positive control (0.1 fmol) is not always detected in any condition, and the gain in sensitivity made by using more RNA for hybridization makes up for the minor increase in background.

**Table 1:** Transcript counts increase as a linear function of total RNA mass used in hybridization.

	Input RNA			
	No RNA	0.1 µg	1 µg	10 µg
Average positive controls	5628	5628	5628	5628
Average negative controls	22	25	25	55
Average targets	21	241	2266	23733
Sum counts	172114	200548	461784	3231759

Average counts of positive and negative control probes, target probes, and the sum of counts over all probes are presented for different masses of total RNA used in hybridization. Data have been normalized by positive control counts.

**Table 2:** The number of genes detected above background increases as more RNA is used in hybridization.

	Input RNA			
	No RNA	0.1 µg	1 µg	10 µg
Positive controls (n = 10)	10	10	9	9
Negative controls (n = 8)	0	0	0	0
Targets (n = 43)	1	23	37	42

The number of genes detected above background is presented for the set of positive controls, negative controls, and targets. Background was modeled for each mass of RNA as the average of negative control transcript counts plus three standard deviations resulting in cutoff values of 43, 49, 49, and 108 counts for no RNA, 0.1 µg, 1 µg and 10 µg, respectively.

Precision was improved by increasing RNA mass used in hybridization. With no RNA, target probes behaved like negative control probes both in terms of transcript counts and coefficient of variation (Figure 2B). Positive and negative control probes were unaffected by no RNA vs. 1 µg, but with 10 µg RNA negative controls produced more counts and a lower coefficient of variation (Table 1; Figure 2B–E), consistent with non-specific interactions contributing to background. The major effect of increasing RNA mass was to increase the number of counts and decrease the coefficient of variation, indicating increases in sensitivity and precision. The median target coefficient of variation was 8.5%, 4.5% and 1.8% for 0.1 µg, 1 µg and 10 µg RNA, respectively. Since technical replicates of a single RNA preparation were used, this experiment captured only technical error, and biological replicates will be more variable. Nevertheless, the decrease in coefficient of variation observed indicates that, with this code set and in this system, the power to detect differential expression is greater when hybridizing more RNA.

Relative transcript abundances were comparable when different masses of RNA were used for hybridization. When comparing results of 0.1 µg and 10 µg RNA, skew in target counts is evident on the low end of transcript abundance, but there is linear concordance for moderate and high abundance transcripts (Figure 2F). This result suggests that the lowest transcript counts (∼10–100) are affected by background, including those detected above background. Minor skew in the same count range is also evident when comparing 0.1 µg and 1 µg, but it is not evident when comparing 1 µg and 10 µg (Figure 2G,H). These results suggest that with this code set and RNA preparation the linear dynamic range of the assay is between 1 µg and 10 µg total RNA, a range that is also optimal for sensitivity and precision. These results also suggest that the system does not reach saturation with 3 million total counts. We conclude that for this code set and this system using microgram quantities of total RNA for hybridization is optimal in terms of sensitivity and precision, and we used 3 µg in subsequent experiments.

We recommend that other researchers working with the nCounter platform perform a similar technical experiment varying the mass of RNA used for hybridization to optimize their results. RNA is often limiting, and such results will aid in considering trade-offs between starting material quantity and data quality. In addition, researchers should consider transcript abundances when possible while designing code sets, since the total number of counts limits sensitivity. For example, the inclusion of invariant genes as internal controls (“housekeeping” genes for normalization) is advisable, but abundantly transcribed genes limit overall sensitivity.

### Specificity of nCounter hybridization

**Figure 3:**
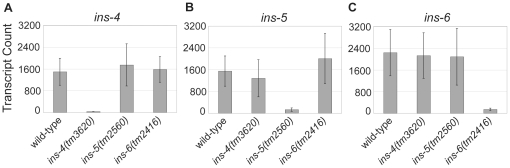
Deletion alleles demonstrate specificity of nCounter hybridization. Average and standard deviation (3 biological replicates) of transcript counts detected during L1 arrest is plotted for *ins-4* (A), *ins-5* (B), and *ins-6* (C) in 4 different strains.

We used deletion mutants to determine the specificity of the nCounter platform with our optimized hybridization conditions (3 µg total RNA). The code set includes 8 negative control probes, but the sequences are from *A. thaliana* and *D. melanogaster* and are less likely to cross-hybridize with *C. elegans* transcripts than the target probes. Because our targets include a 40 gene family and we increased the mass of RNA per hybridization, cross-hybridization is a concern. We obtained deletion alleles *ins-4(tm3620)*, *ins-5(tm2560)* and *ins-6(tm2416)* from the National BioResource Project and prepared RNA for L1 arrest. Deletions *tm3620* and *tm2560* eliminate all of the sequence targeted by the nCounter probes, and *tm 2416* eliminates all but 25 bp. Because the nCounter relies on two probes for each target, *tm2416* should be null in the assay. Each deletion resulted in a dramatic reduction in the number of detected counts, but residual counts were detected (Figure 3). Deletion reduced *ins-4* expression from around 1500 to 31+/−2 counts, *ins-5* was reduced from around 1500 to 124+/−55 counts, and *ins-6* was reduced from around 2000 to 137+/−53 counts. Deletion of *ins-4* is the only one of the three that resulted in a comparable number of counts to the *A. thaliana* negative control probes (Table 1). The results for *ins-5* and *ins-6* suggest that a higher threshold should be used to define background than the one determined from the negative control probes. We tested only three targets for specificity with deletion alleles, and we assume their behavior is representative of the other 37 insulin-like genes. Based on these results, 400 counts is an appropriate background cut-off with this code set using 3 µg RNA. This number corresponds to the max of the three counts after gene deletion plus four times the standard deviation.

### Insulin-like gene expression during the *C. elegans* life cycle

**Figure 4:**
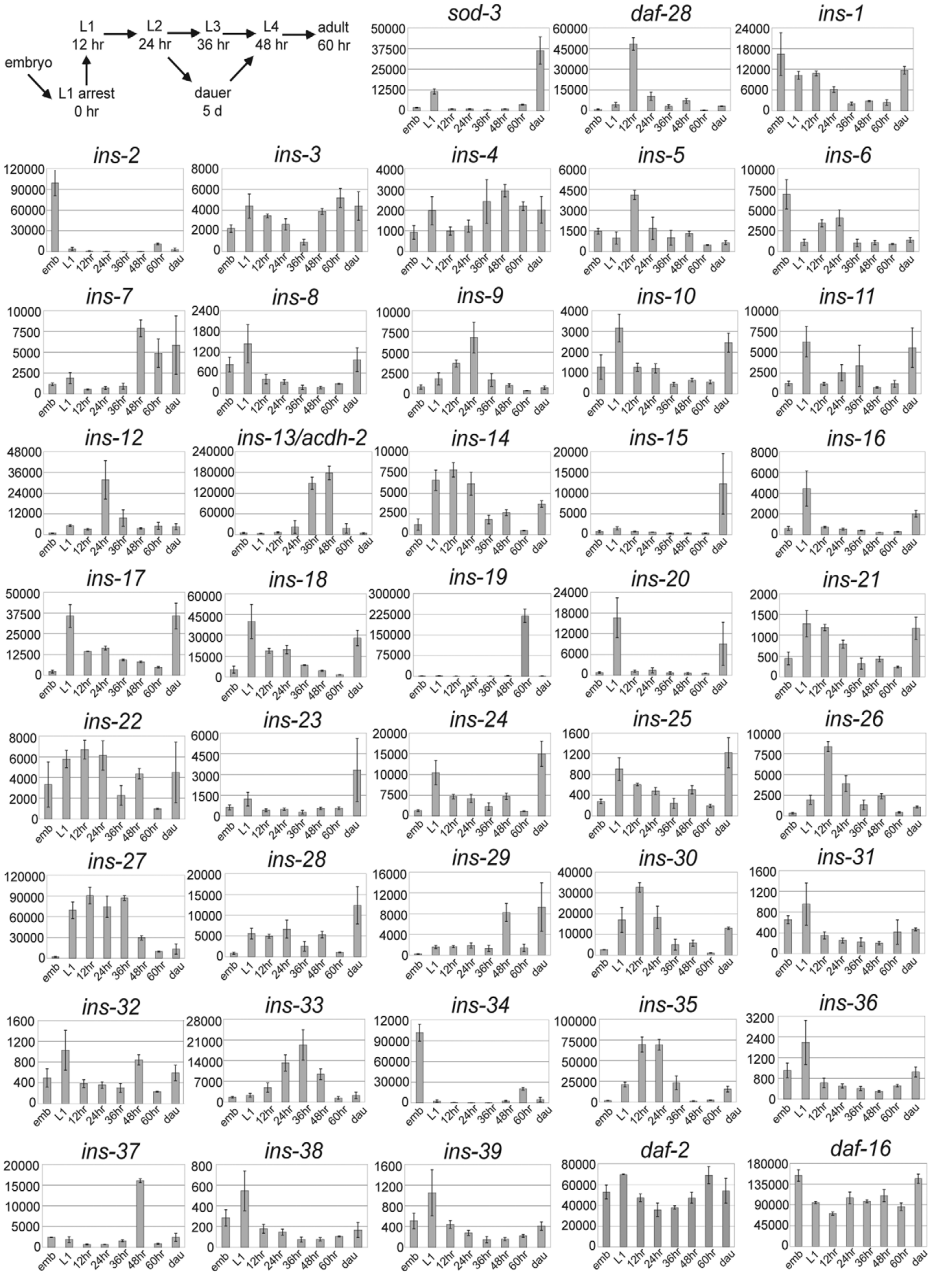
Quantification of insulin-like gene expression during the *C. elegans* life cycle reveals distinct expression patterns. A schematic of the *C. elegans* life cycle is presented along with plots of the average and standard deviation (3 biological replicates) of transcript counts for each gene. The *ins-13* probe is not specific and reports expression of *acdh-2*. In the plots, “emb” refers to mid-grastrulation embryos, “L1” refers to L1 arrest, and “dau” refers to dauer arrest.

We used the nCounter platform to measure insulin-like mRNA expression throughout the *C. elegans* life cycle. mRNA expression was measured from embryos, each larval stage, adults, and during L1 and dauer developmental arrest (Figure 4). The embryos spanned mid-gastrulation. The 60 hr time point includes adults as well as their early embryos. The larval time points (12–48 hr) fall near the end of each larval stage, but because larval stages vary in length, time points fall in variable places within each larval stage and molt cycle. Given the dynamics of development, the samples measured here represent discontinuous developmental stages. Nevertheless, this experiment presents an overview of insulin-like mRNA expression during the life cycle, and future work with high-resolution time series analysis at particular stages will capture true dynamics.

The expression patterns we detected are consistent with published results. Unfortunately, the *ins-13* probe is not specific since the *ins-13* and *acdh-2* genes overlap and share common transcript sequences, and this probe was apparently dominated by *acdh-2* expression [17]. The superoxide dismutase gene *sod-3* is a direct target of DAF-16 [18] known to be up-regulated during L1 arrest and dauer formation [17], [19], [20]. We found that *sod-3* expression is up-regulated by approximately 10-fold and 30-fold during L1 and dauer arrest, respectively, compared to developing larvae (Figure 4). In addition, *daf-28* expression is greatest at the end of L1 development (12 hr), consistent with reporter gene analysis [21]. These results extend on those presented in Figures 1 and 3 to suggest that the data are valid.

Expression of insulin-like genes that have been functionally characterized reveals correlation between function and expression, suggesting that expression patterns can guide functional analysis. For example, expression of *daf-28* during late L1 development is consistent with it promoting bypass of dauer formation given that this is the critical time for the dauer decision [22]. In contrast, *ins-18* is thought to function as an antagonist of the insulin-like receptor DAF-2 [13], [14], and it is up-regulated during L1 arrest and in dauer larvae consistent with promoting developmental arrest. *ins- 10*, *-15*, *-16*, *-17*, *-20*, *-24* have a similar expression pattern, suggesting they could also promote arrest. *ins-7* functions in adults limiting lifespan [14], and it is up-regulated at the end of larval development and in adults (Figure 4). *ins-19* appears to be expressed specifically in adults (60 hr), suggesting that it too functions in adults. However, this time point also includes early embryos that have not yet been laid (earlier embryos than those included in the mid-gastrulation “embryo” sample), and *ins-19* could be expressed maternally or during early embryonic development. *ins-33* is regulated by the heterochronic pathway such that it is repressed during L1 arrest and early larval development, and it promotes germline proliferation, which occurs later in larval development [23], [24]. Consistent with these functional insights, *ins-33* expression increased through larval development, peaking at 36 hr after L1 arrest (Figure 4). *ins-9*, *-35* and others were also expressed during larval development, but with different timing than *ins-33* or *daf-28*, suggesting novel larval functions. There is no known function of insulin-like signaling during *C. elegans* embryogenesis, though the null phenotype of the *daf-2* insulin-like receptor is embryonic lethal [25], [26]. Consistent with a possible function of insulin-like signaling during embryogenesis, and suggesting candidate peptides, *ins-2* and *ins-34* were expressed specifically in embryos (Figure 4).

**Figure 5:**
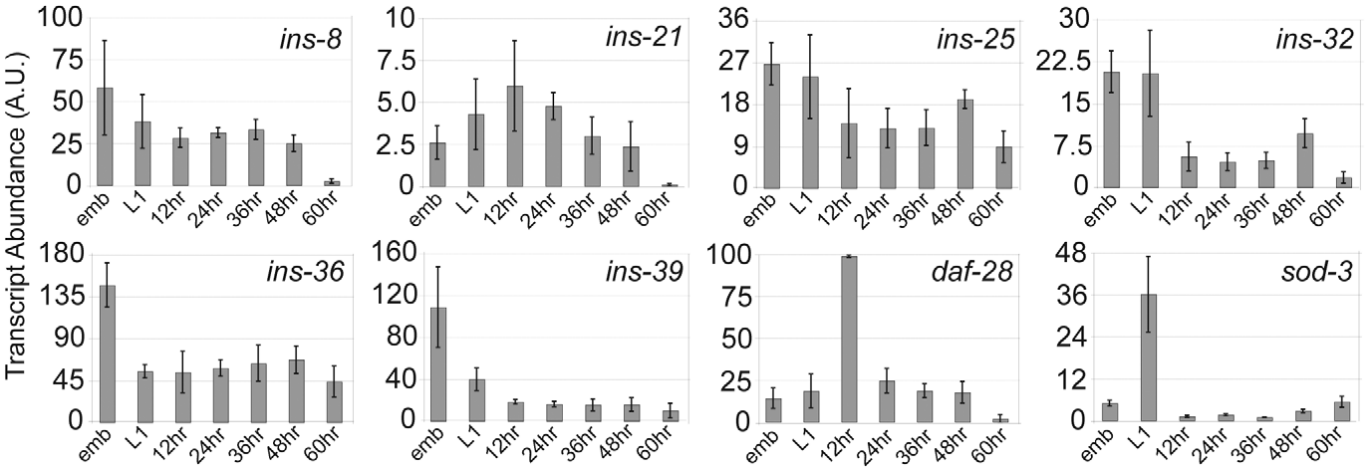
QPCR results in expression profiles similar to nCounter. Average and standard deviation (3 biological replicates) of transcript abundance determined by QPCR is plotted for *ins-8*, *-21*, *-25*, *-32*, *-36*, *-39*, *daf-28* and *sod-3*.

Expression of a few insulin-like genes is very close to the background inferred from deletion analysis (∼400 counts, Figure 3) and should be treated with caution. We used QPCR on the remaining RNA to generate similar expression profiles (excluding dauer since no RNA remained) for a few insulin-like genes with low signal (*ins-8*, *-21*, *-25*, *-32*, *-36* and *-39*) as well as *daf-28* and *sod-3*. *sod-3* and *daf-28* agree remarkably well between platforms (Figure 5). Much of the expression of the 6 low abundance genes is at or below background, but where they are above background there is generally good agreement with QPCR. Nevertheless, there are a couple of examples of what appears to be differential expression between embryo and L1 arrest on one platform not captured on the other. These are at most 2–3-fold differences in expression. Given the low abundance of these transcripts and the modesty of these differences this result does not undermine our nCounter analysis, but it does highlight the need for caution in interpreting results at or near background.

In summary, expression of nearly all 40 insulin-like genes was convincingly detected above background, and expression of most of them was modulated during the life cycle (Figure 4). We did not observe any correlation between expression pattern and classification of peptides as either α, β, or γ based on predicted structural features [13]. Widespread modulation of expression is consistent with extensive developmental and physiological regulation of insulin-like gene expression. The expression patterns are largely distinct, suggesting that regulation of insulin-like gene expression is complex. Furthermore, distinct expression patterns suggest specificity in insulin-like gene function that merits functional analysis.

## Methods

### Nematode culture and sample preparation

RNA samples used for validation were prepared as described [17], and the same RNA was used here for nCounter analysis after 1.5 yr storage at −80°C. The same general procedure was used to prepare RNA for optimization and life cycle analysis, but with less precise staging. Nematode cultures were maintained, passaged and collected at 20°C. A starved 5 cm plate was used to inoculate a 10 ml liquid culture (S-complete medium plus 40 mg/ml *E. coli* HB101), and the liquid culture was incubated for 65 hr at 180 rpm and then bleached to produce a clean preparation of embryos [27]. 500,000 embryos were suspended in 85 ml of S-complete and they were cultured for 31 hr at 180 rpm allowing them to hatch and enter L1 arrest. Cultures were fed by adding HB101 to a final concentration of 40 mg/ml and 5 larvae/µl. Cultures were bleached 60 hr after feeding. Embryos were suspended at 5 eggs/µl in S-complete with no food. The embryo sample was aged 3 hr (to mid-gastrulation) and collected. The remainder of the culture was incubated so that the animals hatched and entered L1 arrest. The L1 arrest sample was collected 24 hr after bleaching, and then the remainder of the culture was fed with 40 mg/ml HB101 to initiate post-embryonic development. Samples were collected 12, 24, 36, 48 and 60 hr later. For the dauer culture, embryos were also suspended at 5 eggs/µl and allowed to hatch and enter L1 arrest. 24 hr after bleaching they were fed with 1 mg/ml HB101 to initiate recovery with limiting food. Dauers were collected after 5 d; cultures had at least 99% dauers based on visual inspection. The entire procedure was repeated 3 times to produce 3 biological replicates. Samples were flash frozen in liquid nitrogen, and RNA was prepared using TRIzol (Invitrogen) plus sand. RNA concentration was determined by UV absorbance and RNA integrity was confirmed by agarose gel electrophoresis. This project has been reviewed and approved by the Duke University Institutional Biosafety Committee (IBC registration# 09-6093-01).

### nCounter analysis

The nCounter code set was designed by NanoString, Inc. (Seattle, WA USA; http://www.NanoString.com/). It includes a pair of approximately 50 nt probes complementary to adjacent sequences in each target transcript. Probes were designed to be specific to the target transcript and to have a uniform melting temperature [15]. Transcript sequences targeted are available in File S1. The *ins-13* probe is not specific, and it appears to report expression of *acdh-2* based on microarray analysis [17]. The code set also includes probes for 10 positive control targets, and those target transcripts were included directly in the code set at known concentrations. The code set also includes 8 probes for negative control genes (from *A. thaliana*). For validation (Figure 1) 0.1 µg total was used for hybridization. For optimization (Figure 2), varying amounts of a single total RNA preparation was used for hybridization (no RNA, 0.1 µg, 1 µg or 10 µg); replicates were performed on a single RNA preparation (technical as opposed to biological). For life cycle analysis, 3 µg total RNA was used per hybridization, and three biological replicates were performed. Hybridization, flow cell preparation and scanning were performed according to the standard nCounter protocol. Transcript counts were normalized between samples in a particular experiment by the positive control transcript counts. For validation and life cycle analysis, target transcript counts were further normalized by the sum of target transcript counts. Ideally, the code set should include genes with invariant expression as internal controls for normalization, but the code set used here does not. The complete data set of insulin-like gene expression during the *C. elegans* life cycle is available in File S2. Microarray analysis was performed as described [17] and the complete data set is available from the Gene Expression Omnibus (GSE11055). Our results are MIAME compliant.

### QPCR analysis

QPCR was performed using the Fast Start Universal SYBR Green Master (Roche) on an Eppendorf MasterCycler QPCR machine according to the manufacturer's instructions. Analysis of QPCR products by gel electrophoresis and melting curves is consistent with amplification of a single, specific product. Genomic DNA was used as template for standard curves for each primer pair, and the standard curve was used to convert cycle thresholds to copy number. cDNA was prepared from total RNA using oligo-(dT) primer and SuperScript III (Invitrogen), and the product was divided between QPCR reactions so that each 20 µl reaction had 20 ng-equivalents of total RNA as template. *rpl-12* and *rpl-19* were used as standards for normalization between RNA/cDNA preparations. Primer sequences used for QPCR are available in File S3.

## Supporting Information

File S1 Complete code set design, including genes, accession numbers, targeted region and target sequence for each gene (including positive and negative controls).
(XLS)

File S2 Complete data set for analysis of insulin-like mRNA expression through the *C. elegans* life cycle. The file contains three sheets: one with raw data, one with the averages of replicates after normalization, and one with the corresponding standard deviations.
(XLS)

File S3 Primer sequences used for QPCR.
(XLS)
